# Evaluating the Role of Tobacco Stalk Biochar in Wheat Growth Under Microplastic Exposure

**DOI:** 10.3390/plants14233578

**Published:** 2025-11-23

**Authors:** Suhang Li, Qiong Yang, Longcheng Jiang, Jiaxin Yao, Yang Luo, Rou Ma, Jiaojiao Liu, Jun Ren, Yangzhou Xiang, Ying Liu

**Affiliations:** 1 School of Geography and Resources, Guizhou Education University, Guiyang 550018, China; 2 School of Biological Sciences, Guizhou Education University, Guiyang 550018, China

**Keywords:** polyethylene microplastic, tobacco stalk-derived biochar, soil remediation, partial least squares path modeling, cascading effects

## Abstract

The accumulation of microplastics in agricultural soils poses a serious threat to both crop production and ecosystem health. To explore potential remediation strategies, we conducted a two-factor pot experiment (PE-MPs × TSB). This study was designed to systematically analyze the interactive effects of polyethylene microplastics (PE-MPs) and tobacco stalk-derived biochar (TSB) on soil properties, physiological characteristics, and growth indicators of wheat. Results indicated that TSB addition significantly increased soil pH, organic matter, and available potassium content, which was associated with a mitigation of the soil acidification and nutrient imbalance observed under PE-MPs. Physiologically, TSB was linked to higher activities of antioxidant enzymes (SOD and POD) and maintained leaf chlorophyll content and photosynthetic function, thereby consistent with a reduction in oxidative stress and better maintenance of growth in the presence of PE-MPs. Furthermore, partial least squares structural equation modeling (PLS-SEM) supported a hypothetical cascading pathway for TSB’s dominant influence: soil improvement → physiological mitigation → growth recovery. The total effect of TSB on biomass (0.71) was substantially greater than that of PE-MPs (0.01). This study proposes a conceptual model and provides correlative evidence that is consistent with multi-level processes through which TSB may alleviate PE-MPs stress, thereby providing theoretical support for the resource utilization of agricultural waste and the green remediation of microplastic-contaminated soil.

## 1. Introduction

The persistent accumulation of microplastics (MPs) in agricultural ecosystems poses a severe challenge to global food security and soil health. These synthetic polymer particles (<5 mm) originate primarily from the fragmentation of agricultural mulch films, wastewater irrigation, and sludge application. Polyethylene dominates this contamination due to its extensive use and resistance to biodegradation, making it one of the most prevalent and persistent contaminants in farmland soils [1,2]. Multiple risks are posed to the soil–plant system by MPs: soil aggregate structure is disrupted, water and nutrient transport are affected, and the composition and function of microbial communities are altered [3,4]. For crops, this contamination translates into a series of abiotic stresses. In wheat (*Triticum aestivum* L.), a staple crop for over one-third of the global population, exposure to PE-MPs has been extensively documented to inhibit seed germination, retard root elongation, impede water and nutrient uptake, and crucially, trigger excessive production of reactive oxygen species (ROS), leading to oxidative damage, Chlorophyll degradation, and ultimately, reductions in biomass and yield [5,6,7].

Concurrently, the search for sustainable agricultural remediation strategies has positioned biochar at the forefront of soil amendment research [8,9,10]. This carbon-rich material, produced through the pyrolysis of biomass under oxygen-limited conditions, is renowned for its porous structure, high specific surface area, and abundant functional groups [11,12]. These properties endow it with a remarkable capacity to improve soil physicochemical properties, such as water retention capacity, cation exchange capacity (CEC), and acidity amelioration, while simultaneously enabling carbon sequestration and adsorption of various organic and inorganic contaminants [13,14]. The properties and functionality of biochar are highly dependent on its feedstock. Given that tobacco straw is an abundant agricultural waste, its conversion into biochar is regarded as an efficient pathway to unlock its potential value [15,16]. The transformation of this waste stream not only provides a practical solution for waste management but also typically yields an alkaline amendment rich in potassium, making it particularly suitable for ameliorating acidic soils and mitigating plant stress [17,18]. Although the efficacy of biochar in alleviating stress from heavy metals [19,20] and organic contaminants [21,22] has been extensively documented, its specific interactions with microplastic contaminants, particularly within wheat production systems, remain an emerging and underexplored field [23]. Most existing studies on microplastic toxicity have employed simplified systems [24], often overlooking the more complex interactions within the soil–plant system. A holistic approach integrating soil nutrient dynamics, key health indicators, and comprehensive plant phenotypic and physiological analyses is therefore essential to unravel the mechanistic pathways through which amendments like biochar confer resilience [25,26].

Therefore, this study was designed to systematically evaluate the role of tobacco stalk-derived biochar (TSB) in influencing wheat growth and soil health under polyethylene microplastic (PE-MPs) stress. Based on existing literature and research objectives, we proposed three interconnected hypotheses, forming a logical chain from soil environment to plant physiology and ultimately to growth performance: (H1) TSB would directly improve soil health by raising pH, increasing organic matter content, and enhancing nutrient availability—particularly available potassium—thereby creating a more favorable growth medium that partially counteracts the physical and chemical disruptions associated with PE-MPs. (H2) The TSB-amended improvement in soil conditions (H1) would subsequently enhance the physiological status of wheat plants under stress, manifested by a strengthened antioxidant defense system (elevated activities of superoxide dismutase (SOD) and peroxidase (POD)), a consequent reduction in oxidative damage (lowered malondialdehyde (MDA) content), and the maintenance of photosynthetic capacity (higher Chlorophyll content). Ultimately, (H3) the TSB-induced improvements in soil properties (H1) and plant physiological status (H2) would synergistically translate into a mitigation of microplastic-induced growth inhibition, resulting in significantly greater plant height and biomass accumulation.

The scope of this investigation is centered on documenting these agronomic and physiological responses and, crucially, on conceptually exploring the potential pathways connecting them. We hypothesize that the observed effects are consistent with a cascade: H1 (soil improvement) → H2 (physiological mitigation) → H3 (growth recovery), which may be initiated by a direct interaction between TSB and PE-MPs (e.g., via adsorption). It is important to note that our approach tests the statistical plausibility of this conceptual model using partial least squares structural equation modeling (PLS-SEM), rather than providing direct experimental verification of the underlying chemical or physical mechanisms. This framework allows us to evaluate not only if TSB is effective, but also to explore how its influence might propagate through the soil–plant system.

From a theoretical standpoint, this research aims to move beyond simply cataloging microplastic phytotoxicity by statistically modeling how TSB amendments are associated with changes in the soil–plant system under stress. Through the combined examination of soil properties and plant physiological and growth parameters, along with the application of statistical modeling techniques, specifically partial least squares structural equation modeling (PLS-SEM), an integrated framework was developed to explore the relationships and test a conceptual model linking contaminants, soil enhancers, and cultivated plants. On a practical level, this study suggests the potential for repurposing tobacco straw, a substantial agricultural byproduct, into a beneficial asset for sustainable land management, thereby advancing the principles of a circular economy. The findings could inform agricultural administrators considering environmentally friendly strategy to address microplastic contamination in farmland soils, thus contributing to efforts aimed at safeguarding of crop productivity and food security in the face of increasing plastic pollution.

## 2. Materials and Methods

### 2.1. Experimental Materials

The soil samples used in this study were collected from Pianpo Village, Wudang District, Guizhou Province (106°47′ E, 26°38′ N). The soil was classified as yellow earth. The topsoil (0–20 cm depth) was obtained, and after plant residues and gravel were removed, it was air-dried, passed through a 2 mm sieve, and homogenized for subsequent use. The initial soil properties were characterized as follows: pH 4.89; organic matter 19.01 g kg^−1^; available nitrogen 36.11 mg kg^−1^; available phosphorus 21.14 mg kg^−1^; available potassium 60.72 mg kg^−1^. The wheat cultivar used was Jimai 22. The PE-MPs were in powdered form of low-density polyethylene. The powder had a particle size of ~100 mesh (approximately 150 μm) and was procured from Chuanguuo Plastic Chemical Co., Ltd. (Dongguan, China). For the PE-MPs, the density was approximately 0.92 g cm^−3^, and no surface functionalization was applied. The TSB was produced by Guizhou Jinyefeng Agricultural Technology Co., Ltd. (Bijie, China) via slow pyrolysis at 380 °C for 2 h under limited oxygen conditions. Key characteristics of the TSB included: pH 9.18; specific surface area 1.47 m^2^ g^−1^; elemental composition (C: 48.3%, H: 3.93%, O: 31.4%, N: 1.50%); and total/available potassium contents of 2.44% and 161.9 mg kg^−1^, respectively [27]. While not quantitatively analyzed in this study, the relatively low pyrolysis temperature (380 °C) typically minimizes the formation of persistent organic pollutants, such as polycyclic aromatic hydrocarbons (PAHs), in TSB. Furthermore, the feedstock (tobacco stalk) is not a known significant source of heavy metals.

### 2.2. Experimental Design

A pot experiment was conducted to investigate the individual and combined effects of PE-MPs and TSB on soil properties and wheat growth. A two-factor completely randomized design was employed, consisting of nine treatments with three replicates each, resulting in a total of 27 pots. PE-MPs were added at three concentrations: 0%, 0.5%, and 1% (*w*/*w*), while TSB was applied at three rates: 0%, 2%, and 4% (*w*/*w*), with all treatments prepared using a 500 g soil matrix. The specific treatments were designed as follows: (1) CK (control, no amendments); (2) M0B1 (2% TSB); (3) M0B2 (4% TSB); (4) M1B0 (0.5% PE-MPs); (5) M1B1 (0.5% PE-MPs + 2% TSB); (6) M1B2 (0.5% PE-MPs + 4% TSB); (7) M2B0 (1% PE-MPs); (8) M2B1 (1% PE-MPs + 2% TSB); and (9) M2B2 (1% PE-MPs + 4% TSB). All pots were randomly arranged and cultivated in a greenhouse.

The concentrations of PE-MPs (0.5% and 1% *w*/*w*) were selected based on previous pot studies investigating microplastic phytotoxicity in crops [5,6], which often employ these levels to elicit measurable physiological responses within short timeframes. These concentrations are higher than the average levels currently found in most agricultural soils but are representative of heavily contaminated hotspots, such as fields with long-term plastic mulch use or sludge application. These concentrations were chosen to simulate a high-stress scenario, enabling a clear evaluation of biochar’s mitigating potential, rather than to reflect typical global background concentrations.

The experiment was conducted in a greenhouse at Guizhou Education University in October 2024. Precisely weighed amounts of PE-MPs, TSB, and soil mixtures (with a total mass of 500 g) for each treatment were thoroughly homogenized and then transferred into plastic pots (15 cm upper diameter, 8 cm lower diameter, 9 cm height). The soil moisture was adjusted to 60% of the field water capacity and stabilized for 7 days. Subsequently, uniformly plump wheat seeds were selected, surface-sterilized in 75% ethanol for 30 min, rinsed with sterile water, soaked for 12 h, and finally sown at a depth of 2 cm. The germination rate was determined 7 days after sowing according to the method described by Du et al. [24]. After 30 days of growth, both wheat seedlings and soil samples were collected for subsequent analysis.

### 2.3. Analysis of Soil Physicochemical Properties

Soil physicochemical properties were determined according to the methods described by Bao [28]. The specific indicators and procedures were as follows: (1) Soil pH was measured potentiometrically. Water-soluble or exchangeable hydrogen ions were extracted from the soil using deionized water or a neutral salt solution, and the potential difference of the extract was measured with a pH glass electrode–calomel electrode system, and the pH value was directly recorded. (2) Soil organic matter (SOM) was determined by the potassium dichromate external heating method. Under strongly acidic conditions, excess potassium dichromate was used to oxidize soil organic carbon, and the remaining dichromate was titrated with ferrous sulfate, and the organic carbon content was calculated based on the consumption, and then multiplied by the empirical coefficient of 1.724 to obtain the SOM content. (3) Alkaline-hydrolyzed nitrogen (AN) was measured by the alkali hydrolysis diffusion method. In a sealed diffusion dish, soil samples were reacted with sodium hydroxide solution to release ammonium nitrogen, which was absorbed by a boric acid absorption solution; the absorbed nitrogen was then titrated with a standard hydrochloric acid solution, and the alkaline hydrolyzed nitrogen content was calculated based on the acid consumption. (4) Available phosphorus (AP) was extracted with sodium bicarbonate and determined by molybdenum-antimony anti-colorimetry. Soil was extracted with a 0.5 mol L^−1^ sodium bicarbonate solution (pH 8.5), and phosphate in the extract reacted with molybdenum-antimony anti-reagent to form phosphomolybdenum blue, and its absorbance was measured at 700 nm wavelength, and the available phosphorus content was calculated from a standard curve. (5) Available potassium (AP) was extracted with ammonium acetate and determined by flame photometry. Soil was extracted with a 1 mol L^−1^ ammonium acetate solution (pH 7.0), and the extract was appropriately diluted before the potassium ion emission intensity was measured with a flame photometer, and the available potassium content was quantified using an external standard method.

### 2.4. Analysis of Growth and Physiological Traits in Wheat Seedlings

A systematic analysis of physiological indicators was performed on wheat plants employing the following protocols: (1) Plant height was measured with a steel tape from the soil surface to the highest natural point of the plant. (2) For biomass determination, plant samples were oven-dried to constant weight at 80 °C after de-enzyming at 105 °C for 30 min. The aboveground dry weight was subsequently measured using a precision analytical balance. (3) Chlorophyll content was determined by homogenizing 0.2 g of fresh leaf tissue with a small amount of quartz sand, calcium carbonate powder, and 2–3 mL of pre-cooled 95% anhydrous ethanol in an ice bath until the tissue turned white. The homogenate was incubated for 3 min, filtered, and diluted to 25 mL with the same ethanol. Absorbance was measured at 665 nm and 649 nm using a UV spectrophotometer, and Chlorophyll content was calculated. (4) Peroxidase (POD) activity was assayed by homogenizing 0.5 g of leaf tissue with 4 mL of pre-cooled extraction buffer in an ice bath, followed by centrifugation at 10,000× *g* for 20 min at 4 °C. The resulting supernatant was collected and stored at −20 °C. POD activity was determined using a commercial assay kit (Cat. No. R30312, Shanghai Yuanye Bio-Technology Co., Ltd., Shanghai, China) according to the manufacturer’s instructions. (5) Superoxide dismutase (SOD) activity was measured by homogenizing 0.4 g of leaf tissue with 1 mL of pre-cooled extraction buffer in an ice bath. The homogenate was rinsed with 3 mL of extraction buffer, transferred to a centrifuge tube, adjusted to a final volume of 4 mL, and centrifuged at 4000× *g* for 20 min at 4 °C. The supernatant was maintained on ice prior to analysis. SOD activity was determined using the corresponding kit (Cat. No. R22261) (Bio-Technology Co., Ltd., Shanghai, China) following the manufacturer’s protocol. (6) To quantify malondialdehyde (MDA) content, 0.4 g of leaf tissue was homogenized in 4 mL of buffer on ice. After centrifugation at 4000× *g* for 10 min, the supernatant was collected. MDA levels were then determined using an assay kit (Cat. No. R21874) (Bio-Technology Co., Ltd., Shanghai, China) as per the manufacturer’s protocol.

### 2.5. Statistical Analysis

A two-way analysis of variance (ANOVA) was performed to determine the main effects of biochar (BC) and polyethylene microplastics (PE-MPs), as well as their interactive effect, on all soil properties, plant physiological characteristics, and growth indicators. Significant differences between treatment means were assessed using the least significant difference (LSD) test at a 95% confidence level (*p* < 0.05). Graphs were generated using OriginPro 2025b software (OriginLab Corporation, Northampton, MA, USA). Data in the column graphs are presented as means ± standard deviation (n = 3). Mantel tests with 999 permutations were performed to evaluate the relationships between wheat growth characteristics and soil properties, using the ‘LinkET’ package [29]. To overcome the limitations of correlation analysis in assessing relationships among multiple factors simultaneously, we employed partial least squares structural equation modeling (PLS-SEM) with the “plspm” package [30]. PLS-SEM was conducted to assess the direct and indirect effects of TSB, PE-MPs, plant physiology (PP), soil properties (SP), and plant height (PH) on seedling biomass. All statistical analyses were performed using R statistical software (version 4.3.3; R Core Team, Vienna, Austria).

## 3. Results

### 3.1. Effects of TSB on Soil Physicochemical Properties Under PE-MP Contamination

All treatments significantly altered soil pH relative to the control (*p* < 0.05; Table 1). A pronounced increasing trend was observed with increasing TSB application rates under consistent PE-MP concentrations, demonstrating TSB’s efficacy in enhancing soil alkalinity and mitigating acidification in the yellow earth soil. Although individual PE-MPs treatments (M1B0, M2B0) exhibited elevated pH compared to the control, pH showed an initial increase followed by a decrease as the PE-MP concentration rose PE-MPs concentration, indicating that while TSB substantially enhanced soil pH, PE-MPs exerted comparatively modest effects. Concurrently, TSB amendment significantly influenced organic matter content, where excluding M1B0 and M2B0 treatments showing no significant difference (*p* > 0.05), all other treatments exhibited substantial enhancements (*p* < 0.05), particularly under constant PE-MPs concentrations where organic matter content progressively increased with TSB application, reaching a maximum of 120.04 g kg^−1^ in M2B2 treatment, equivalent to an 85.5% increase over the control.

Regarding nutrient availability (Table 1), alkaline hydrolyzed nitrogen showed significant variations among all treatments (*p* < 0.05) except M2B2, with response patterns varying by pollution level: initial increase followed by reduction without PE-MPs, progressive decline at 0.5% MP concentration, and ascending trend at 1% MP concentration, culminating in the highest value of 75.04 mg kg^−1^ in M1B0 treatment, representing a 53.8% increase. Similarly, available phosphorus content remained largely unchanged in most treatments (*p* >0.05) except M1B2, M2B1 and M2B2, generally increasing with TSB amendment across microplastic conditions through steady increases in non-polluted and 0.5% MP treatments versus initial rise followed by reduction at 1% MP concentration, with the maximum value of 36.31 mg kg^−1^ documented in M1B2 treatment, corresponding to an 80.65% increase. Furthermore, available potassium content displayed significant improvements in all treatments (*p* < 0.05) except M1B0 and M2B0, consistently enhancing with increasing TSB application rates across all microplastic concentrations and ultimately reaching a peak value of 114.15 mg kg^−1^ in M2B2 treatment, which represents an 86.12% improvement compared to the control.

As detailed in Table 2, two-way ANOVA revealed that TSB had significant main effects on soil pH, SOM, AN, and AK (all *p* < 0.05), with an exceptionally strong influence on SOM (F = 5367.650, *p* < 0.001). PE-MPs also showed significant main effects on SOM and AN (*p* < 0.01), but no significant effects on AP or AK. Significant interactive effects between TSB and PE-MPs were observed for SOM and AN (both *p* < 0.001), indicating that the amendment efficacy of biochar on soil nutrients was modulated by microplastic contamination levels.

### 3.2. TSB Amendment Modulates Wheat Physiological Traits Under PE-MP Stress

**Figure 1:**
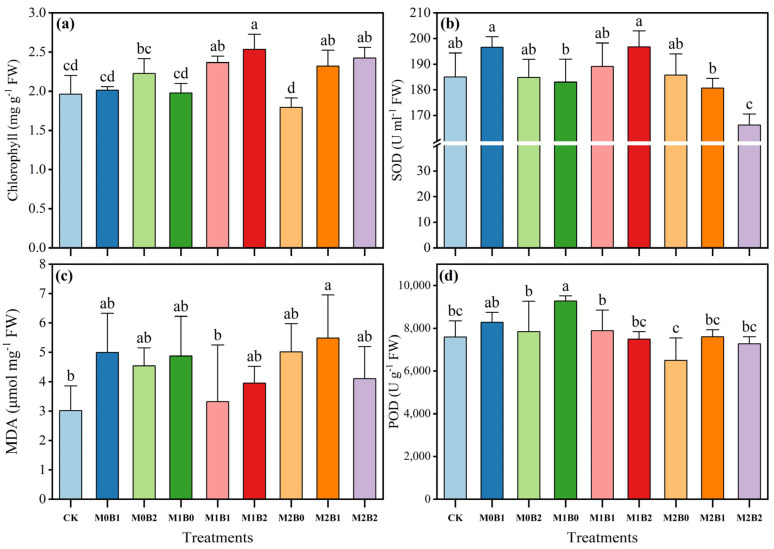
Effects of TSB on physiological characteristics of wheat under PE-MPs contamination. Error bars represent standard deviation (SD) (n = 3). (**a**) Chlorophyll content; (**b**) SOD content; (**c**) MDA content; (**d**) POD content. Different lowercase letters in captions denote significant differences at the 0.05 level among treatments.

No significant changes in Chlorophyll content were observed in TSB-alone treatments (M0B1, M0B2) without microplastic contamination compared to the control (CK) (*p* > 0.05; Figure 1a). This result indicates that TSB amendment alone had limited effects on Chlorophyll synthesis in wheat. Under MP contamination conditions, differential Chlorophyll responses were observed among the combined treatments. The lowest Chlorophyll content (1.79 mg g^−1^) was recorded in M2B0, whereas TSB addition restored Chlorophyll levels, demonstrating partial alleviation of MP-induced inhibition of Chlorophyll synthesis and photosynthetic capacity. Data from Figure 1b indicate that except for M2B2 showing significant difference from the control (*p* < 0.05), no significant variations were observed among other treatments (*p* > 0.05). Under identical MP concentrations, SOD activity exhibited concentration-dependent responses to TSB: an initial increase followed by decrease without MPs; progressive increase at 0.5% MP; and decreasing trend at 1% MP. The maximum SOD activity (196.72 U mL^−1^) was achieved in M1B2, corresponding to a 6.32% increase over the control. The results presented in Figure 1c show that except for M2B1 demonstrating significant difference (*p* < 0.05), MDA content remained unchanged in other treatments (*p* > 0.05). Under fixed MP concentrations, MDA content displayed distinct patterns with TSB addition: initial rise then declines without MPs; decrease followed by increase at 0.5% MP; and increase followed by decrease at 1% MP. The highest MDA content (5.49 μmol mg^−1^) was measured in M2B1, indicating a 45.05% increase compared to the control. As evidenced in Figure 1d, POD activity showed significant difference only in M1B0 versus the control (*p* < 0.05), with no significant changes in other treatments (*p* > 0.05). Under equivalent MP conditions, POD activity exhibited varying trends with TSB application: initial increase then decrease without MPs; gradual decrease at 0.5% MP; and initial increase followed by decrease at 1% MP. The peak POD activity (9276.44 U g^−1^) was recorded in M1B0, representing a 22.14% enhancement relative to the control.

The two-way ANOVA results presented in Table 3 demonstrate that PE-MPs had significant main effects on Chlorophyll content, SOD, and POD activities (*p* < 0.05), whereas TSB exerted a particularly strong significant main effect only on Chlorophyll content (F = 21.467, *p* < 0.001). Significant interactive effects between PE-MPs and TSB were observed for SOD and POD activities (*p* < 0.05), indicating that the alleviation of microplastic-induced oxidative stress by biochar was influenced by pollution levels. Critically, MDA content was not significantly affected by PE-MPs, TSB, or their interaction (all *p* > 0.05), which clearly indicates that lipid peroxidation, as measured by MDA, was not a statistically altered endpoint under the present experimental conditions. Therefore, under the present experimental conditions, the mitigation of oxidative stress by TSB is more closely correlated with the enhancement of enzymatic antioxidant activities (SOD and POD). The lack of a significant increase in MDA content across treatments suggests that membrane lipid peroxidation was not a pronounced endpoint of oxidative damage in this study.

### 3.3. Effects of TSB on Growth of Wheat Under PE-MP Contamination

**Figure 2:**
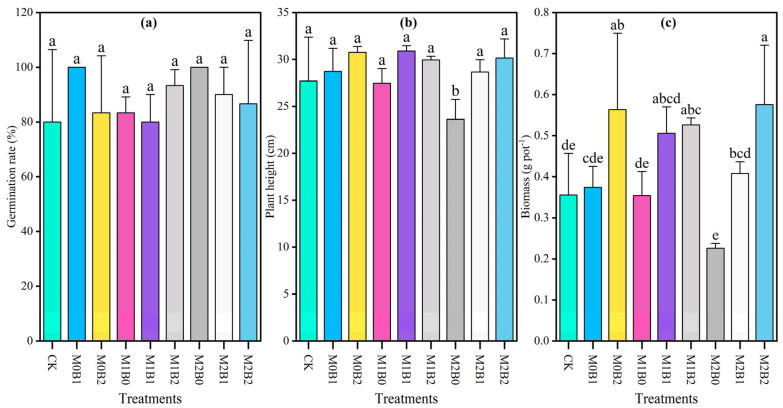
Effect of TSB on the growth of wheat seedlings exposed to PE-MPs. Error bars represent standard deviation (SD) (n = 3). (**a**) Germination rate; (**b**) Plant height; (**c**) Biomass. Different lowercase letters in captions denote significant differences at the 0.05 level among treatments.

TSB application under microplastic contamination conditions had no significant effect on wheat germination rate (*p* > 0.05, Figure 2). Plant height was significantly reduced by 1.48% in the high-concentration microplastic treatment (M2B0) compared to the control, indicating pronounced inhibitory effects of 1% microplastic pollution. In combined treatments, plant height showed recovery compared to microplastic-only treatments, demonstrating that TSB amendment effectively alleviated these inhibitory effects. Both TSB-alone and combined treatments produced higher biomass than the control, with biomass increasing with higher TSB application rates at each microplastic concentration. Combined treatments consistently exhibited greater biomass than corresponding microplastic-only treatments, indicating that TSB can partially mitigate the negative impacts of PE-MPs on wheat biomass accumulation. Significant differences were detected between M0B2, M1B2, and M2B2 treatments and the control (*p* < 0.05), while no significant differences were observed among remaining treatments (*p* > 0.05).

According to the two-way ANOVA results summarized in Table 4, TSB exhibited significant main effects on plant height and biomass (*p* < 0.01), whereas PE-MPs had no significant independent effects on any growth parameters. No significant interactive effects were observed for all growth indicators (*p* > 0.05), demonstrating that the growth-promoting effects of biochar remained consistent across different microplastic stress levels. Germination rate was unaffected by any treatment, supporting the hypothesis that the early seedling growth stage is more sensitive to microplastic stress than seed germination.

### 3.4. Biotic and Abiotic Drivers of Wheat Phenotypic Traits

**Figure 3:**
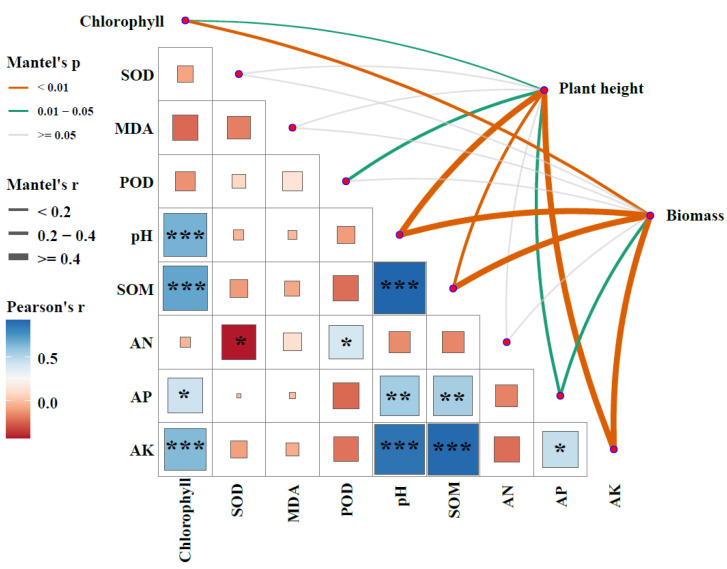
Relationships among wheat traits (plant height and seedling biomass), physiological indices (Chlorophyll content; SOD, MDA, and POD activities), and soil properties (pH, SOM, AN, AP, AK). Edge thickness represents the magnitude of Mantel’s r, while color indicates statistical significance. Pairwise correlations between variables are colored according to Pearson’s correlation coefficients. Asterisks (*, **, ***) represent statistical significance at the *p* < 0.05, *p* < 0.01, and *p* < 0.001 levels, respectively. The size of square indicates the absolute value of the correlation coefficient (Mantel r statistic) between two variables.

**Figure 4:**
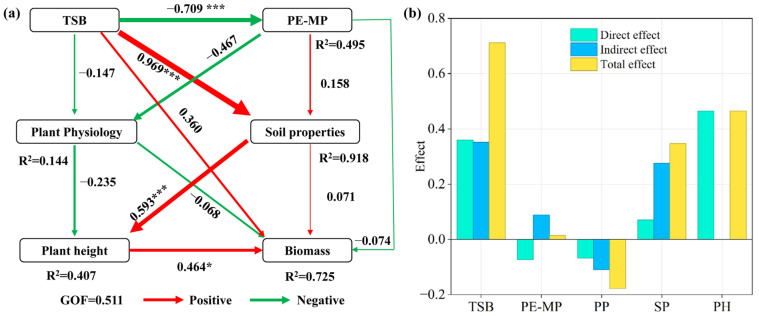
Results of partial least squares structural equation modeling (PLS-SEM). (**a**) Path model illustrating the effects of TSB amendment and PE-MPs on seedling biomass. Path coefficients are numerically displayed, with line thickness representing effect magnitude and asterisks denoting statistical significance (* *p* < 0.05, *** *p* < 0.001). R^2^ values indicate the variance explained for each endogenous variable. (**b**) Direct and indirect effects of TSB, PE, plant physiology (PP), soil properties (SP), and plant height (PH) on seedling biomass.

The Mantel test was used to assess the associations between wheat traits (plant height and seedling biomass) and a suite of physiological indicators (Chlorophyll content; SOD, MDA, and POD activities) as well as soil properties (pH, SOM, AN, AP, AK) across the nine treatments (Figure 3). Results indicated that soil pH, SOM, and AK were significantly correlated with plant height (*p* < 0.01). Similarly, Chlorophyll content, soil pH, SOM, and AK showed significant correlations with seedling biomass. Furthermore, plant height was influenced by Chlorophyll content, POD activity, and AP content, whereas seedling biomass was affected by AP content (*p* < 0.05). The partial least squares structural equation model (PLS-SEM) explained 72.5% of the variation in seedling biomass (Figure 4a), indicating strong predictive capacity. The path coefficient from TSB to PE-MPs was −0.709 (*p* < 0.001), which is statistically consistent with the hypothesis that TSB significantly alleviates microplastic stress on wheat seedlings through a direct interaction (e.g., adsorption). Although TSB exhibited no significant direct effect on seedling biomass, it exerted a positive indirect influence by significantly improving soil properties and promoting plant height, which consequently enhanced biomass accumulation. In contrast, the effects of PE on seedling biomass, whether direct or indirect through altering soil properties or plant physiology, were not statistically significant (*p* > 0.05). Among all direct influencing factors, including TSB, PE, plant physiology (PP), soil properties (SP), and plant height (PH), only plant height showed a significant positive effect (*p* < 0.05) on seedling biomass. Total effect analysis further revealed that TSB (0.71) had a substantially stronger overall influence on seedling biomass compared to PE (0.01) (Figure 4b).

## 4. Discussion

This study systematically investigated the ameliorative effects of TSB on PE-MPs-contaminated soil and its promotional effect on wheat growth, and all three initial hypotheses (H1, H2, H3) were fully validated. TSB was demonstrated to significantly improve wheat growth indicators under microplastic stress through the amelioration of soil physicochemical properties, enhancement of antioxidant capacity, and promotion of photosynthetic performance. The discussion will therefore be structured around three main aspects: modifications in soil properties, plant physiological responses, and integrated mechanistic pathways. Based on the findings, both the limitations of the current study and potential future research directions will also be proposed.

### 4.1. TSB Ameliorates Soil Properties Under PE-MP Stress

Our results fully support hypothesis (H1), indicating that tobacco stalk-derived biochar (TSB) effectively counteracted the adverse soil conditions induced by PE-MPs. The primary improvement was a marked increase in soil pH from 4.89 to 6.30 in the high-dose TSB treatment (M2B2, Table 1). This shift from strong acidity to near-neutrality, likely driven by the dissolution of alkaline components in TSB [31,32], is critical for mitigating aluminum toxicity and improving nutrient availability. Concurrently, TSB amendment significantly enhanced soil organic matter (SOM) and available potassium (AK) by up to 85.5% and 86.12%, respectively. These changes collectively point to TSB’s role in creating a more favorable rhizosphere environment—not only by directly supplying carbon and potassium but also by potentially improving soil structure and cation exchange capacity [33,34]. We propose that this multi-faceted improvement in soil physicochemical properties forms the foundational stage for alleviating plant stress.

### 4.2. TSB Modulates Wheat Physiological Responses to PE-MPs

The physiological data reveal that TSB did not simply suppress the plant’s stress response but modulated it in a context-dependent manner, providing nuanced support for hypothesis (H2). The significant interactive effects on SOD and POD activities (Table 3) suggest that TSB primed the antioxidant system, enabling a dynamic defense that was calibrated to the level of PE-MPs stress [35,36]. For instance, the highest SOD activity was recorded in the M1B2 treatment, indicating a potentiated response at moderate stress. Crucially, the absence of significant changes in malondialdehyde (MDA) content across treatments suggests that oxidative damage to membranes was not a major factor under our experimental conditions. Therefore, TSB’s primary role appears to be the enhancement of functional resilience—bolstering the capacity of the enzymatic antioxidant system to prevent damage, rather than repairing it after the fact.

This physiological resilience extended to photosynthesis. Under high PE-MPs stress (1%), TSB amendment (4%) significantly restored chlorophyll content, indicating a mitigation of photosynthetic inhibition. This recovery may be linked to the improved magnesium nutrition from the biochar [37] and/or the better overall water and nutrient status facilitated by the improved soil environment [38]. In summary, TSB helped maintain physiological homeostasis under stress by reinforcing antioxidant defenses and preserving photosynthetic function.

### 4.3. An Integrated Hypothetical Pathway: Linking Soil Improvement to Growth Recovery

Our findings that TSB improves soil properties (Section 4.1) and modulates plant physiology (Section 4.2) collectively support hypothesis (H3), showing mitigated growth inhibition. To synthesize these observations into a coherent narrative, we employed Partial Least Squares Structural Equation Modeling (PLS-SEM). The model (Figure 4a) allows us to propose a hypothetical cascade pathway to explain TSB’s dominant influence on biomass.

The PLS-SEM analysis indicates that the strong total effect of TSB on biomass (0.71) was primarily realized through indirect routes, a finding that aligns with the emerging consensus that biochar often functions as a soil-conditioning “ecosystem engineer” rather than a direct plant growth stimulant [39,40]. The model is consistent with a three-step process: (1) TSB first directly and robustly improves soil properties (path: TSB → SP = 0.795 ***), fulfilling H1. This is a well-documented effect of biochar, attributed to its ability to amend acidity, add stable carbon, and release nutrients [31,41]. (2) The improved soil environment subsequently supports enhanced plant physiological status (SP → PP = 0.461 *), for instance by providing essential co-factors like potassium for antioxidant enzymes [36], and directly promotes morphogenesis, as seen in plant height (SP → PH = 0.624 *), fulfilling H2. The strong link between soil properties and plant height underscores the importance of root zone conditions for early plant development [42]. (3) Finally, these improvements collectively contribute to greater biomass accumulation (PH → Biomass = 0.585 *), demonstrating how foundational improvements cascaded through the system to yield the final growth outcome.

A key finding from the model is the strong negative path from TSB to PE-MPs (TSB → PE-MPs = −0.709 ***). This provides statistical support for the hypothesis of a direct interaction (e.g., adsorption or passivation), which we propose as a plausible initiating event that reduces the effective stress exposure of the plant [43,44]. However, it is critical to state that this interaction was not directly measured in this study. Therefore, we posit that growth recovery is an emergent property of a reconditioned soil–plant system, driven by a cascade of improvements from the soil upwards, rather than by a single, isolated mechanism.

### 4.4. Limitations and Future Perspectives

This study has several limitations that should be acknowledged. First, while our PLS-SEM analysis suggested a direct interaction between TSB and PE-MPs (path coefficient: –0.709), this remains a statistical inference lacking direct experimental validation, such as through adsorption isotherms or microscopic examination (e.g., SEM-EDS). Second, the 30-day pot experiment may not capture the long-term dynamics of biochar and microplastic aging in soil. Finally, the use of a single type of powdered PE-MPs and tobacco stalk-derived biochar in an acidic yellow earth limits the generalizability of our findings, as the remediation efficacy likely varies with microplastic polymer types, physical forms, biochar feedstocks, and soil properties.

Future research should prioritize addressing these limitations through three key directions: (1) Employing laboratory adsorption experiments coupled with spectroscopic and microscopic techniques to directly quantify and characterize the interaction between TSB and PE-MPs; (2) Evaluating the temporal evolution of these interactions and their consequences for crop yield and soil health under realistic agricultural conditions; and (3) Investigating how biochar’s efficacy is modulated by its inherent properties (e.g., feedstock, pyrolysis temperature), the type and form of microplastics, and contrasting soil environments to develop tailored remediation strategies.

## 5. Conclusions

This study provides evidence that TSB is associated with enhanced wheat growth in microplastic-contaminated soil, which may be attributed to a general improvement of soil fertility (elevating pH, organic matter, and available potassium), alongside an apparent modulation of the physiological oxidative response linked to PE-MP exposure through complex interactions within the soil–plant system. Our findings suggest that the role of TSB could be dual in nature, functioning both as a general soil conditioner and a potential resilience enhancer. Statistical modeling (PLS-SEM) supported our proposed hypothetical cascade for this recovery, initiating from soil amelioration, in which soil mitigation is linked to improved physiological status (enhanced antioxidant defense and maintained photosynthetic performance), which in turn is correlated with growth promotion. Future studies are needed to experimentally confirm each step of this pathway. Consequently, the conversion of tobacco stalk into biochar presents a dual-value strategy. It not only offers a practical pathway for the resource utilization of agricultural waste but also shows potential as a sustainable amendment for remediating microplastic-contaminated farmland.

## Figures and Tables

**Table 1:** Impact of TSB on soil physicochemical properties under PE-MPs.

Treatments	pH	SOM (g kg^−1^)	AN (mg kg^−1^)	AP (mg kg^−1^)	AK (mg kg^−1^)
CK	4.87 ± 0.08 d	17.94 ± 1.17 g	34.65 ± 11.49 b	20.10 ± 9.66 d	61.33 ± 2.29 e
M0B1	5.73 ± 0.13 b	46.83 ± 1.92 f	36.06 ± 7.90 b	24.29 ± 2.50 bcd	95.31 ± 2.86 cd
M0B2	6.25 ± 0.10 a	89.82 ± 2.52 c	23.52 ± 5.64 b	29.09 ± 2.16 abcd	105.76 ± 5.85 abc
M1B0	5.17 ± 0.09 c	18.09 ± 0.46 g	75.04 ± 3.50 b	21.40 ± 3.77 cd	55.47 ± 12.63 e
M1B1	5.76 ± 0.08 b	82.70 ± 2.37 d	36.07 ± 2.34 b	26.67 ± 2.94 abcd	89.56 ± 6.12 d
M1B2	6.29 ± 0.06 a	112.95 ± 1.66 b	23.67 ± 5.58 b	36.31 ± 6.88 a	106.51 ± 4.61 ab
M2B0	5.03 ± 0.05 c	18.45 ± 0.40 g	32.25 ± 9.38 b	30.00 ± 10.12 abcd	53.23 ± 6.20 e
M2B1	5.75 ± 0.07 b	74.33 ± 1.18 e	33.48 ± 13.05 b	34.91 ± 3.85 ab	96.49 ± 8.16 bcd
M2B2	6.30 ± 0.10 a	124.04 ± 3.11 a	65.53 ± 12.09 a	32.75 ± 11.27 abc	114.15 ± 0.54 a

Note: Values represent mean ± standard deviation (n = 3). Different lowercase letters within the same index indicate significant differences among treatments (*p* < 0.05).

**Table 2:** Two-way ANOVA of soil physicochemical properties as affected by TSB and PE-MPs.

Variables	PE-MPs		TSB		PE-MPs × TSB	
	F	P	F	P	F	P
pH	4.810	0.021	493.765	0.000	2.462	0.082
SOM	355.021	0.000	5367.650	0.000	106.988	0.000
AN	6.686	0.007	4.912	0.020	19.835	0.000
AP	3.153	0.067	3.822	0.041	0.779	0.553
AK	1.100	0.354	157.083	0.000	1.371	0.283

**Table 3:** Two-way ANOVA of wheat seedling physiological characteristics as affected by TSB and PE-MPs.

Variables	PE-MPs		TSB		PE-MPs × TSB	
	F	P	F	P	F	P
Chlorophyll	4.490	0.026	21.467	0.000	1.990	0.139
SOD	8.043	0.003	1.776	0.198	4.865	0.008
MDA	1.205	0.323	0.273	0.764	2.134	0.118
POD	4.906	0.020	0.602	0.558	3.092	0.042

**Table 4:** Two-way ANOVA of wheat seedling growth as affected by TSB and PE-MPs.

Variables	PE-MPs		TSB		PE-MPs × TSB	
	F	P	F	P	F	P
Germination rate	0.483	0.625	0.069	0.934	1.440	0.262
Plant height	2.119	0.149	8.726	0.002	1.236	0.331
Biomass	0.909	0.421	15.543	0.000	1.444	0.260

## Data Availability

The original contributions presented in this study are included in this article. Further inquiries can be directed to the corresponding author.
